# Dissociating premotor and motor components of response times: Evidence of independent decisional effects during motor-response execution

**DOI:** 10.3758/s13423-025-02663-z

**Published:** 2025-03-07

**Authors:** Saman Kamari Songhorabadi, Simone Sulpizio, Michele Scaltritti

**Affiliations:** 1 Dipartimento Di Psicologia e Scienze Cognitive, Università Degli Studi Di Trento, Corso Bettini 31, 38068 Rovereto, TN Italy; 2 Dipartimento Di Psicologia, Università Degli Studi Di Milano-Bicocca, Milan, Italy; 3 Milan Center for Neuroscience (Neuromi), Università Degli Studi Di Milano-Bicocca, Milan, Italy

**Keywords:** Mental chronometry; response time; decision making; motor-response execution

## Abstract

Traditional measures of response times (RTs) capture the summed duration of multiple latent and overt processes, including motor-response execution. The present research assessed the functional independence of the decisional components unfolding before vs after the onset of the muscular activation in the context of a lexical decision task requiring manual button-press responses. Specifically, the lexicality effect (slower latencies for nonwords compared to words) was separately tracked across premotor and motor components of RTs under different regimes of decision bias. Whereas at the premotor level the lexicality effect was modulated by the proportion of word vs nonword trials in the block, with a reversal of the lexicality phenomenon when nonwords occurred in 75% of the trials, motor times (i.e., a chronometric measure of response duration) consistently displayed longer durations for nonword responses, irrespective of bias manipulation. The results point to a partial functional independence between the decisional components involved at the premotor vs motor level, suggesting that the onset of motor behavior may represent the onset of specific decisional processes, rather than the termination or the continuation of computations unfolding in the premotor interval.

**Supplementary Information:**

The online version contains supplementary material available at 10.3758/s13423-025-02663-z.

## Introduction

Mental chronometry, one of the earliest breakthroughs for modern psychological sciences, builds on the notion that response time (RT) captures the duration of the multiple processes involved in a task (e.g., Luce, 1986; Posner, 1978), including the motor-activity required to perform the overt response. In case of discrete behavioral responses typically used in psychological experiments (e.g., button-press), RTs can in fact be divided into a premotor time (PMT), extending from stimulus onset to the initiation of motor activity, and a motor time (MT), extending from the onset of motor activity until the completion of the response (Botwinick & Thompson, 1966; Weiss, 1965). The functional characterization of the MT, as well as its functional relationship with PMT, remain contentious to this day.

Traditionally, the onset of motor behavior has been considered the endpoint of upstream cognitive computations and the beginning of motor-response execution, envisaged as a separate stage within serial architectures (e.g., McClelland, 1979; Ratcliff, 1978; Sternberg, 1969). Under this perspective, PMTs and MTs can be mapped onto independent categories of processes represented, respectively, by central cognitive vs peripheral motor computations. This view has been challenged by different lines of evidence. Pioneering research has highlighted online processes of response control operating during the unfolding of the motor-response itself (e.g., Allain et al., 2004; Burle et al., 2002; Fluchère et al., 2018; Hasbroucq et al., 1999; Ramdani et al., 2013, 2021; Roger et al., 2014), mainly through inhibitory and corrective mechanisms on muscular activations. Minimally, motor-response execution is thus shaped by monitoring processes related to executive and cognitive control. In addition, results from perceptual decision-making tasks indicate that the amount of sensory evidence exerts a reliable influence on both PMTs and MTs (Dendauw et al., 2024; Servant et al., 2021; Weindel et al., 2021), pointing to the accumulation of a decision variable that propagates beyond response onset and informs motor-response execution (e.g., Calderon et al., 2018; Dendauw et al., 2024; Eriksen & Schultz, 1979; Verdonck et al., 2021). Under this perspective, the cognitive characterization of MTs is largely inherited from PMTs, with the two intervals being shaped by the same cognitive/decisional factor(s) that continuously unfold over both components.

A further set of findings, however, seems to expand the hypothesis space by pointing to a differentiation in the decisional phenomena that modulate premotor vs. motor intervals, and consequently in the underlying decisional components. First, not all decision-related manipulations propagate their influence from PMT to MT (Weindel et al., 2021), as one would expect under the assumption of a single decisional variable informing both intervals. In particular, studies focusing on the visual lexical decision paradigm, in which participants categorize strings of letters as real words (e.g., *house*) vs nonwords (e.g., *flirp*), have shown that while the lexicality effect (slower RTs for nonwords compared to words) reliably affect both PMTs and MTs, the word frequency effect remains bounded to PMTs (Scaltritti et al., 2023; but see Dendauw et al., 2024). Second, different decision-related phenomena, when jointly manipulated, can combine in different ways across PMTs and MTs. Specifically, lexicality and speed-accuracy tradeoff (SAT) manipulations in lexical decision display interactive effects at the level of PMTs, but additive effects on MTs, with the difference between words and nonwords remaining constant in terms of execution times, irrespectively of the SAT regime (Scaltritti et al., 2024). Taken together, these findings suggest that, although motor-response execution is permeable to multiple cognitive factors, it may not merely reflect the same dynamics observed during PMTs.

The last line of evidence comes from the comparison between correct vs incorrect responses in binary decision tasks, in which error trials display faster PMTs, but slower MTs, compared to correct responses (e.g., Allain et al., 2004; Rochet et al., 2014; Smigasiewicz et al., 2020; Weindel et al., 2021). This peculiar pattern has been mostly related with online response control mechanisms, which would try to inhibit incorrect responses during their unfolding. Importantly, it suggests that PMTs an MTs are empirically dissociable, by showing opposite-going effects across the two intervals.

In this research, we examined the degree of (in)dependence between premotor and motor decisional components by combining a lexical decision task with a bias manipulation. The lexicality effect on RTs is in fact amplified when most trials require a word response, and reversed when most trials require a nonword response (e.g., Wagenmakers et al., 2008). This pattern is used here as a litmus test for the independence between PMTs and MTs. The results reported for RTs may be expected to be reflected both at the level of PMTs (which indeed capture the bulk of the lexicality effect) and MTs. This seems even more likely when considering how a bias toward a given stimulus category affects both decision time and response execution (e.g., Starns & Ma, 2018; Voss et al., 2010).

The prediction is however non-trivial, when considering that the lexicality effect on MTs has proven impervious to manipulations that otherwise influence its magnitude at the premotor level, such as in the case of SAT manipulations (Scaltritti et al., 2024). Motor times can thus display independent lexicality effects compared to those displayed by PMTs. In this scenario, bias-induced modulations in the overall lexicality effect may manifest within the PMT component, while MTs may continue to display a constant lexicality effect, regardless of bias manipulations. Specifically, when the bias is toward words, PMTs (but not MTs) should display an enhanced lexicality effect. More critically, when the bias is toward nonwords, MTs would consistently display a standard lexicality effect even under conditions that either cancel or reverse its premotor manifestation. This dissociation would provide strong evidence of the empirical and functional independence between PMTs and MTs.

To assess this empirical question, we acquired the electromyographic (EMG) signal for the muscle responsible of button-press responses during the lexical decision task. Using single-trial EMG traces, a response-time fractioning technique (Botwinick & Thompson, 1966) was applied to partition each RT into a PMT and a MT. Other than chronometric measures, we assessed the influence of bias on the lexicality effect in terms of multiple indexes of response accuracy, including variations in error rates as a function of response speed (e.g., Gratton et al., 1988; van den Wildenberg et al., 2010), and partial errors, consisting of sub-threshold muscular activation of the response hand associated with the incorrect response (e.g., Eriksen et al., 1985; Hasbroucq et al., 1999). These indexes should qualify variations in the lexicality effect across different conditions of bias, highlighting changes associated with different policies of online response control.

## Method

### Participants

The sample size was determined based on recent guidelines in the field (Brysbaert, 2019) and past studies (Scaltritti et al., 2023, 2024). Forty-eight Italian native speakers participated in the experiment (41 females, *M*_*age*_ = 22.60; *SD*_*age*_ = 3.77). All participants reported having normal or corrected vision, no motor impairments, and no history of neurological issues or learning disabilities. Following the administration of the Edinburgh Handedness Inventory (Oldfield, 1971), 42 participants were classified as pure right-handers (*M* = 88.32, *SD* = 13.60), 2 as pure left-handers (with handedness scores corresponding to − 80 and − 100), 3 as mixed right-handers (*M* = 26.67, *SD* = 20.82), and 1 as a mixed left-hander (− 46.7). Data from 2 participants were discarded either due to low accuracy in the task (i.e., below 2.5 *SD*s from the overall sample mean) or for an excessive proportion of trials that had to be rejected (> 25%) given the noisy signal and the subsequent inaccurate detection of EMG onset. The final sample thus included 46 participants. All the procedures of experiment were approved by the ethical committee of the University of Trento (protocol number 2023–064), and participants signed an informed consent document. Participants were compensated with 20€ or course-credits. Raw data and materials are available at [https://osf.io/v3cx4/].

### Stimuli

Three sets of 120 words each were drawn from the PhonItalia database (Goslin et al., 2014), and 3 sets of 120 nonwords were created. Nonwords consisted of orthographically (and phonologically) legal strings, mostly created by assembling different syllables of the Italian language (i.e., pseudowords). Words and pseudowords within each set (as well as across all sets) were comparable for a series of psycholinguistic variables (Table 1). In addition, 240 filler words and 240 filler pseudowords were selected/created to be used in biased blocks. Fillers and experimental items were comparable across the psycholinguistic properties listed in Table 1. Only experimental items were considered in the analyses, so that the same pseudowords and words, as well as an equal number of trials were analyzed across different blocks. Items within each set, as well as filler items, were further partitioned into 2 subsets for the counterbalancing of response-hand across participants (see below). The resulting subsets were comparable for the variables listed in Table 1.

**Table 1:** Psycholinguistic variables for the 3 sets of stimuli

	Set 1			Set 2			Set 3		
Variables	Words	PWs	t	Words	PWs	t	Words	PWs	t
Frequency (log)	3.71	-	-	3.73	-	-	3.48	-	-
N. of letters	6.84	6.84	0	6.84	6.84	0	6.84	6.84	0
N. of syllables	2.85	2.92	0.84	2.86	2.95	0.95	2.88	2.95	0.75
Orthographic N	1.98	2.04	0.17	1.99	2.03	0.12	1.99	2.03	0.12
OLD20	2.17	2.20	0.38	2.23	2.27	0.47	2.19	2.24	0.64
Bigr. freq. sum	608,475	638,593	0.76	617,306	631,586	0.40	641,558	640,927	0.02
Bigr. freq. mean	102,904	104,640	0.38	103,836	107,481	0.75	108,147	109,354	0.28

PWs = pseudowords; N. of Letters = number of letters; Orthographic N = orthographic neighborhood; OLD20 = orthographic Levenshtein distance to the twenty closest neighbors (Yarkoni et al., 2008); Bigr. freq. sum = summed bigram frequency; Bigr. freq. mean = mean bigram frequency. Words’ variables were extracted from the PhonItalia database (Goslin et al., 2014). For pseudowords, the number of orthographic neighbors and OLD20 were computed with reference to the PhonItalia database using functions from the *vwr* package (Keuleers, 2013) in R. Bigram frequency values were computed using a custom-made script with reference to the same database. Reported *t*-values are from independent samples, two-tailed t-tests

### Apparatus and procedure

Participants first provided demographic and health-related information by completing a questionnaire. After the installation of the EMG electrodes (see EMG recording and processing section), the experiment began. The experimental procedure was administered through the E-Prime 3 software (version 3.0.3.80) running on a laptop. Participants were comfortably seated in front of the screen, at a distance of ~ 50 cm, holding 2 cylindric handheld buttons, one per hand, connected to a Blackbox Toolkit module. They were instructed to categorize letter-strings as words or pseudowords by using their thumbs to perform the corresponding button-press response.

The experiment consisted of 3 main blocks. In the standard condition, words and pseudowords had an equal probability of occurrence (50%; 240 total trials). Differently, in the word-bias condition, 75% of the trials presented a word and 25% of the trials featured a pseudoword stimulus. In the pseudoword-bias condition, these probabilities were reversed (25% of the trials presented a word, 75% a pseudoword). Blocks of the standard condition thus featured 240 trials in total, whereas the biased block included 480 trials (240 of which were fillers). In this way, the same number of experimental trials (i.e., 240) was analyzed across the different bias conditions. Halfway through each block, the stimulus—(word vs pseudoword) response (left- vs right-hand) pairing was reversed, to ensure an equal number of responses from the two hands for each category of stimuli in each condition. The experiment always began with the standard condition, to avoid carry-over phenomena, and the order of the following blocks (word- and pseudoword-bias) was counterbalanced across participants. The assignment of the 3 sets of stimuli to the blocks, as well as the order of alternation of the stimulus–response mapping within each block were counterbalanced across participants. Before each block, and before each inversion of the stimulus–response pairing, 16 practice trials were administered, with the proportion of words and pseudowords following the one featured in the following experimental block. Self-terminated breaks were prompted every 120 trials. The experimental session took about 105 min to complete.

Stimuli appeared in 25-pt Courier New font, written in black on a gray background (RGB = 190, 190, 190). Each trial began with a fixation cross, with a random duration corresponding to 700, 750, 800, or 850 ms. The stimulus was then displayed until response or until the response deadline (1500 ms), after which the stimulus was terminated even if no response had been given. A blank screen lasting 400 ms served as the inter-trial interval. Error and time-out feedback messages (“*ERROR*”; “*TOO SLOW*”) were displayed in red font for 500 ms only during the practice phases. 

### EMG recording and processing

EMG activity from the *flexor pollicis brevis* of both hands was acquired through an eego sports system (ANT Neuro) operating at a 1,000 Hz sampling rate. Two-pairs of disposable bipolar electrodes were placed ~ 2 cm apart on the thenar eminences of both hands, and the ground electrode was placed on the pisiform bone of the right wrist. Before installation, skin preparation of the recording site included the application of isopropyl alcohol and a mildly abrasive gel (Nuprep). EMG recordings were monitored online, and the experimenter(s) asked participants to relax when noise from tonic activity became visible. Offline (pre)processing was conducted using EEGLAB (version 14_1_2b; Delorme & Makeig, 2004) and ERPLAB (Lopez-Calderon & Luck, 2014) functions in MATLAB (version 2018b, MathWorks Inc., Natick, MA, USA), together with custom scripts.

A 10 Hz, order 2 Butterworth high-pass filter and a 50 Hz notch filter were applied to the continuous EMG signals of both hands. Epochs going from −500 until 2,100 ms (with 0 corresponding to stimulus onset) were then segmented. Two custom-made scripts were applied to the epochs. The onset of the response-related EMG activity was detected within each epoch using the integrated profile method devised by Liu & Liu, 2016 (see also Scaltritti et al., 2023; 2024; Weindel et al., 2021). Afterwards, to support the detection of artifacts and partial responses, within each epoch we identified windows of EMG activity in correspondence to those (consecutive) samples in which the rectified EMG signal exceeded the threshold of 3.5 SDs above the mean absolute activity computed in the baseline (i.e., from −500 to 0 ms). Of the resulting windows, those separated by an interval shorter than 25 ms were aggregated into a single one, whereas windows shorter than 50 ms or beginning after the button-press were excluded. The script highlighted all the epochs in which 2 or more windows of activity were detected. All the epochs were visually inspected and retained only when the EMG onset was accurately placed in correspondence to the last window of activity representing the response-related EMG burst, to exclude artifacts stemming from signal drift, noise, and false starts. On average, 1.77% (*SD* = 2.48%) of the trials per participant were rejected following these criteria.

The same processing pipeline was applied to the hand not involved in the button-press, to track partial errors and partial correct responses. Those epochs featuring one or more windows of activity within these channels were marked by the algorithm. Each epoch was then visually inspected and partial responses were considered valid when the covert EMG activation was clustered in a visually clear burst and its onset was accurately detected. Partial errors were found, on average, in 4.75% of the total trials (*SD* = 3.28%). Partial correct responses were sparse (*M* = 0.31%, *SD* = 0.35%) and thus not considered further.

### Measures

#### Chronometric measures

The chronometric analysis focused on pure-correct response (i.e., trials featuring correct responses with no covert activation in the non-response hand) and experimental items. Within each trial, RTs were partitioned into PMTs (from stimulus onset until the onset of the EMG burst) and MTs (the interval between the onset of the EMG burst and the button-press). The 3 measures were separately analyzed.

#### Response accuracy

Accuracy analyses included correct responses and errors, irrespective of the presence of partial EMG activation in the non-responding hand. Trials in which participants failed to respond within the allotted time (0.01%) were instead removed from the analysis. Variations in accuracy as a function of response latency were investigated via conditional accuracy functions (CAFs). Trials were divided into 5 quantiles as a function of RTs and quantiles were then included as a fixed effect in the analyses. Partial errors were analyzed by focusing on correct responses to assess the likelihood of covert incorrect activations across experimental conditions.

### Statistical analyses

Chronometric measures were analyzed with linear mixed-effects (LME) models, whereas indexes of response accuracy were analyzed using generalized mixed-effects models (GLMEs), to accommodate the dichotomous nature of the dependent variables. Within GLMEs, the maximum number of allowed iterations was increased (2^5^) and a *bobyqa* optimization algorithm was used for the second stage optimization of the models. Fixed effects (lexicality: word vs pseudoword; bias: neutral, word-bias, pseudoword-bias; interaction terms) were considered significant when the corresponding *t* or *z* statistics were larger than |2|. We began by fitting the model structure of maximal complexity (Barr et al., 2013), featuring by-participants and by-items random intercepts, as well as random slopes and correlations. Models were progressively simplified by removing random terms associated with 0-variance or, in case of failures to converge, by first removing correlations and then the terms associated with the smallest amount of variance. For CAFs, fixed effects included the interaction between lexicality, bias, and quantiles. Second order polynomials were used to model the quantile variables to accommodate non-linear trends. The random-effect structure was limited to random intercepts to aid convergence. Follow-up comparisons were conducted on estimated marginal means, using a Tukey correction in case of multiple comparisons. Analyses were performed using the *lme4* (version 1.1–33; Bates et al., 2015), the *afex* (1.3; Singman et al., 2021), and the *emmeans* (1.8.6) packages in R (version 4.3.0; R Core Team, 2015). Figures were made through the *ggplot2* package (version 3.4.2; Wickham, 2016) in R.

## Results

### Chronometric measures

**Fig. 1:**
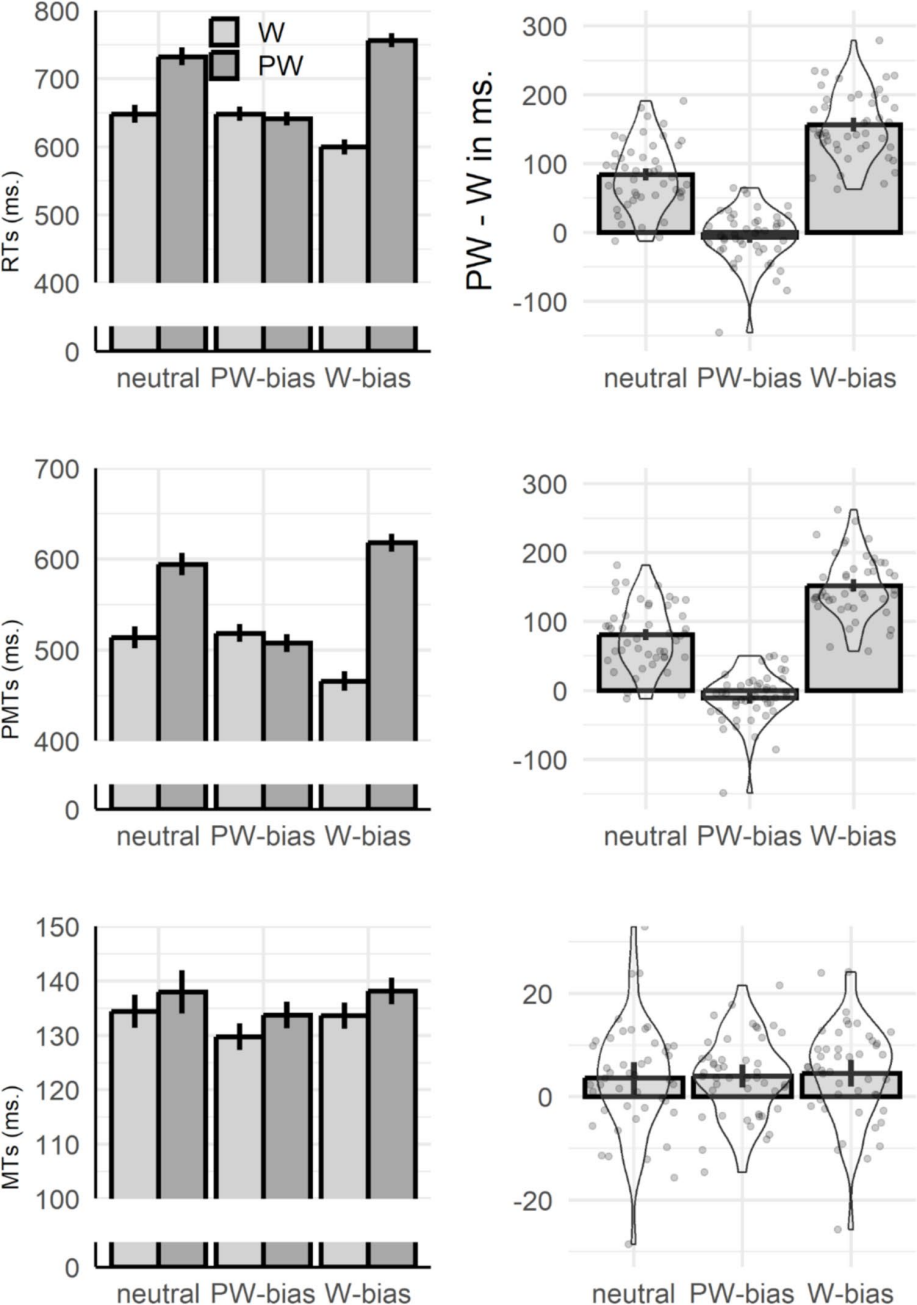
Results for response time (RT; first row), premotor time (PMT; second row), and motor time (MT, third row). The first column reports mean chronometric measures by conditions (W = word; PW = pseudoword). The second column reports the mean lexicality effect (pseudoword – word) as a function of bias conditions. Points represent individual difference scores, and the violin plots describe their distribution. For all plots, error bars display 95% confidence intervals, adjusted for within-participants variables following Morey (2008)

Parameters of the fixed effects are listed in Table 2 (for random effects, see Supplemental Materials 1, Table S1). For RTs, the significant interaction between lexicality and bias highlighted that the classic lexicality effect (words faster than pseudowords) detected in the neutral condition (*Est*. = −83.8, *SE* = 8.21, *z* = −10.20, *p* < 0.001) was canceled when there was a bias towards pseudoword responses (*Est*. = 10.8, *SE* = 1.56, *z* = 1.56, *p* = 0.12), and enhanced when a word-bias was introduced (*Est*. = −159.0, *SE* = 8.24, *z* = −19.29, *p* < 0.001). Similarly, for PMTs the significant lexicality by bias interaction indicated that the standard lexicality effect of the neutral condition (*Est*. = −80.2, *SE* = 7.20, *z* = −11.35, *p* < 0.001) was enhanced when the bias was toward words (*Est*. = −154.3, *SE* = 8.05, *z* = −19.16, *p* < 0.001) and tended to reverse (words slower than pseudowords, *Est*. = 15.1, *SE* = 7.82, *z* = 1.93, *p* = 0.05) when the majority of trials included pseudoword stimuli. In contrast, the interaction between lexicality and bias was not significant on MTs, as confirmed via Bayes Factor (BF) estimation (Raferty, 1995), with BF = 0.0007 signaling strong support for the null hypothesis. In fact, the lexicality effect, with faster MTs for words than pseudowords, was similar under all bias conditions (neutral: *Est*. = −3.51, *SE* = 1.30, *z* = −2.69, *p* = 0.007; pseudoword bias: *Est*. = −4.02, *SE* = 1.85, *z* = −2.17, *p* = 0.030; word bias: *Est*. = −4.53, *SE* = 1.99, *z* = −2.27, *p* = 0.022). In terms of the simple effect of the bias manipulation, MTs were overall faster in blocks where pseudowords occurred in 75% of the trials, compared to neutral conditions (*b* = −4.72, *SE* = 2.08, *t* = −2.27). Results are summarized in Fig. 1.

**Table 2:** Fixed effects for LME models on chronometric measures

	RT			PMT			MT		
Fixed effects	Est	SE	t	Est	SE	t	Est	SE	t
Intercept	652.76	14.03	46.53	518.14	12.96	39.99	134.40	3.58	37.50
Bias (pseudo.)	1.15	7.69	0.15	6.37	6.80	0.94	−4.72	2.08	−2.27
Bias (word)	−51.21	8.41	−6.09	−50.19	6.75	−7.43	−0.81	2.08	−0.39
Lexicality (pseudo.)	83.79	8.21	10.20	80.21	7.20	11.13	3.51	1.30	2.69
Bias (pseudo.) x Lexicality (pseudo.)	−94.57	5.44	−17.37	−95.35	4.88	−19.53	0.50	1.57	0.32
Bias (word) x Lexicality (pseudo.)	75.24	6.71	11.22	74.11	5.25	14.12	1.02	1.73	0.59

*RT* response time, *PMT* premotor time, *MT* motor time, *SE* standard error, *pseudo.* pseudoword

### Response accuracy

In terms of response accuracy, the lexicality by bias interaction (Table 3; for random effects see Table S2) indicated that the non-significant lexicality effect (i.e., words more accurate than pseudowords) in the neutral condition (*Est*. = 0.04, *SE* = 0.15, *z* = 0.30, *p* = 0.76), was significantly enhanced when word responses were biased (*Est*. = 1.49, *SE* = 0.16, *z* = 9.39, *p* < 0.001), whereas it displayed the opposite direction (i.e., pseudowords more accurate than words) when the bias was towards the pseudoword category (*Est*. = −1.89, *SE* = 0.15, *z* = −12.22, *p* < 0.001). A similar pattern surfaced for partial errors (Table 3; for random effects see Table S2). Under neutral conditions, no significant difference surfaced between words and pseudowords in their likelihood to trigger a covert incorrect response (*Est*. = −0.15, *SE* = 0.11, *z* = −1.40, *p* = 0.16). Under biased conditions, partial errors were more likely for the less frequent response category (pseudoword bias: *Est*. = 1.59, *SE* = 0.13, *z* = 11.80, *p* < 0.001; word bias: *Est*. = −1.42, *SE* = 0.12, *z* = −11.58, *p* < 0.001).

**Table 3:** Fixed effects for generalized LME models on accuracy measures

	Accuracy			Partial Errors		
Fixed effects	Est	SE	z	Est	SE	t
Intercept	3.95	0.15	26.31	−3.32	0.15	−22.68
Bias (pseudo)	−1.24	0.10	−12.81	0.68	0.10	6.98
Bias (word)	0.49	0.11	4.29	−0.69	0.12	−5.96
Lexicality (pseudo)	−0.04	0.15	−0.30	0.15	0.11	1.40
Bias (pseudo) x Lexicality(pseudo)	1.94	0.14	13.35	−1.74	0.15	−11.67
Bias (word) x Lexicality (pseudo)	−1.45	0.15	−9.62	1.27	0.14	9.13

*SE* standard error *pseudo*. pseudoword

**Fig. 2:**
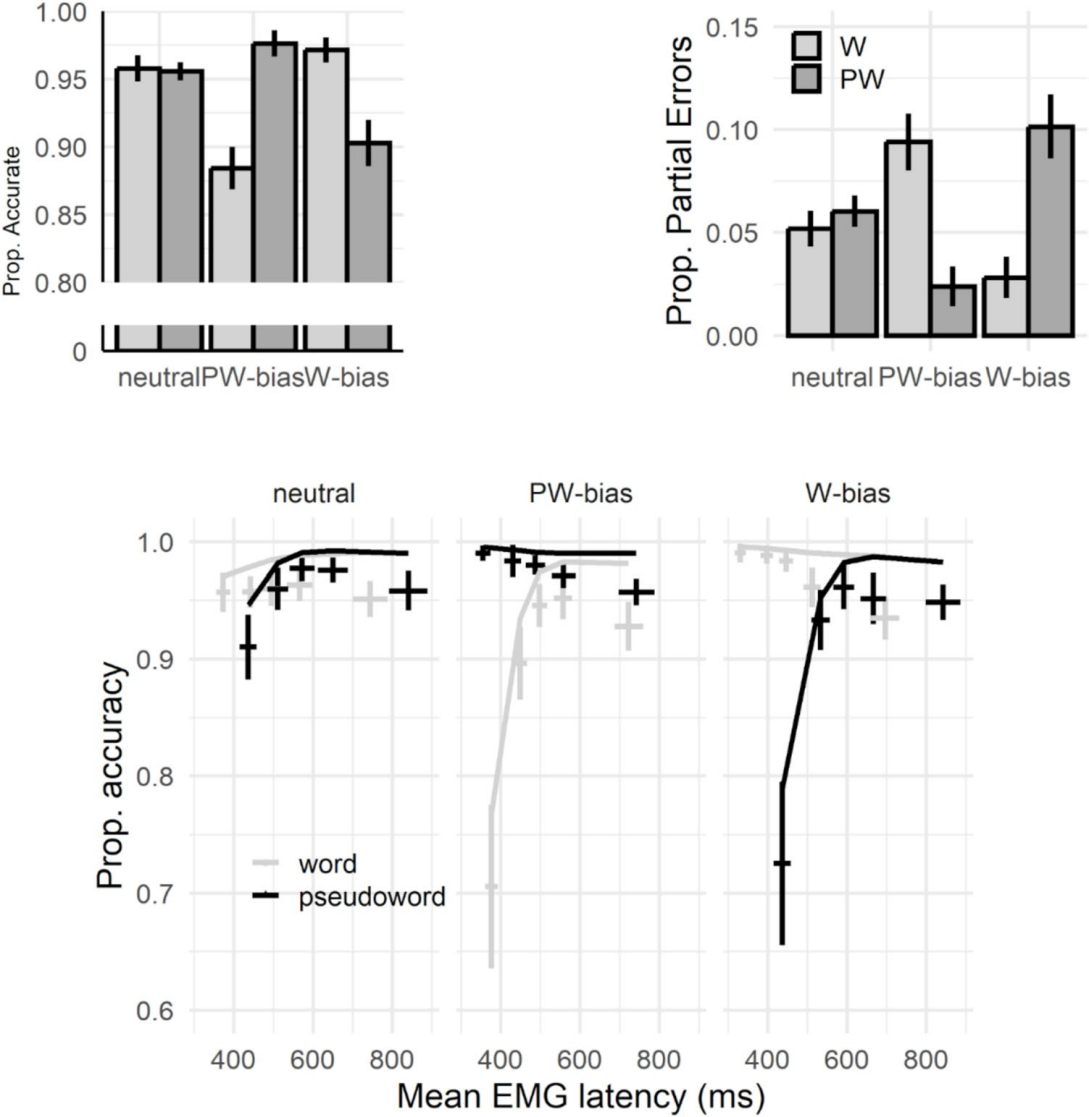
Results for accuracy, partial errors, and conditional accuracy functions. W = word; PW = pseudoword; Prop. = proportion. For CAFs (second row), points represent empirical means, with vertical error bars highlighting 95% confidence intervals. Horizontal error bars reflect 95% confidence interval of the average EMG onset within each quantile (both adjusted for within-participants variables following Morey, 2008). Lines represent models’ predicted means

Within CAFs, the non-linear interaction between quantile, lexicality and bias (Table 4; for random effects, see Table S2) reveled that the standard finding of an increased likelihood of fast errors for pseudowords found under neutral conditions was altered by the bias manipulation. Specifically, impulsive error characterized the more frequent response category. All the results of the accuracy analyses are summarized in Fig. 2.

**Table 4:** Fixed effects for the generalized LME model on CAFs

Fixed Effects	Est	SE	z
Intercept	4.12	0.16	25.30
Bias (pseudo)	−1.03	0.09	−11.60
Bias (word)	0.76	0.11	6.65
Lexicality (pseudo.)	0.06	0.15	0.39
Quantile, linear	77.50	6.74	11.50
Quantile, quadratic	−3.57	5.79	−0.62
Bias (pseudo) X Lexicality (pseudo.)	1.70	0.15	11.38
Bias (word) X Lexicality (pseudo.)	−1.61	0.18	−10.90
Bias (pseudo) X Quantile, linear	98.46	6.36	15.48
Bias (word) X Quantile, linear	−145.46	7.13	−20.40
Bias (pseudo) X Quantile, quadratic	−74.75	7.96	−9.39
Bias (word) X Quantile, quadratic	2.51	9.16	0.27
Lexicality (pseudo.) X Quantile, linear	32.73	7.49	4.37
Lexicality (pseudo.) X Quantile, quadratic	−65.00	6.82	−9.53
Bias (pseudo) X Lexicality (pseudo.) X Quantile, linear	−258.54	8.58	−30.14
Bias (word) X Lexicality (pseudo.) X Quantile, linear	208.69	7.74	26.98
Bias (pseudo) X Lexicality (pseudo.) X Quantile, quadratic	166.95	11.08	15.06
Bias (word) X Lexicality (pseudo.) X Quantile, quadratic	−33.26	9.72	−3.42

*SE* standard error, *pseudo.* pseudoword, *acc.* accuracy

## Discussion

This study investigated the functional independence of information processing between premotor and motor components of RTs by tracking the lexicality effect (difference between nonwords and words) across different conditions of decision (and response) bias within a lexical decision task featuring words and wordlike nonwords (pseudowords). The results revealed that, akin to previous observations (Wagenmakers et al., 2008), the lexicality effect on RTs was magnified when the bias was toward word responses and eliminated when the bias was toward pseudoword responses. At the level of PMTs, the results mimicked those on RTs, while also displaying a tendency towards a reversal of the lexicality effect when the bias was toward pseudoword responses. Importantly, the lexicality effect on MTs, with slower execution times for pseudowords compared to words, remained constant across the three conditions of bias. This pattern indicates a functional dissociation between premotor and motor stages of RTs with respect to the underlying decisional components, as proven by the opposite-going effects elicited by the same decisional phenomenon (i.e., the lexicality effect) across PMTs and MTs. It thus seems that there is a differentiation in the generative processes shaping the effect before and after the boundary of motor-activity onset.

The bias manipulation had a strong impact on the accuracy measures related to online response control, such as fast impulsive and partial errors, confirming an influence at the level of response execution (Starns & Ma, 2018; Voss et al., 2010). Importantly, the lexicality effect on MTs seems to dissociate even with respect to these indexes. In standard lexical decision paradigms, pseudowords are associated not just with slower MTs, but also with lower response accuracy and an increased likelihood of fast impulsive errors, possibly stemming from lexical capture phenomena (Scaltritti et al., 2021, 2023, 2024). These circumstances may prompt a stronger engagement of the response-monitoring system, to detect and correct errors. Consistently, pseudowords seem associated with higher rates of partial errors (Scaltritti et al., 2021, 2023), a phenomenon that maps onto online-response control by empirically capturing the online corrective mechanisms unfolding during response execution. The present experiment reveals that higher rates of both fast-impulsive and partial errors are not necessarily associated with the condition yielding slower MTs. In fact, when pseudowords occurred on 75% of the trials, the rates of both fast-impulsive errors and partial errors were largely superior for words. Even under these circumstances, MTs still remained slower for pseudowords, suggesting that action monitoring, at least with respect to its online response control component, is not the main determinant of the lexicality effect on MTs.

Chronometrically, the simple effect of the bias manipulation on MTs (Table 2) suggests that MTs may have been overall faster within blocks where pseudowords were the majority of the items. It is tempting to speculate that, since pseudowords are characterized by slower execution time, a response bias in their favor may have prompted faster overall MTs across the whole block, including word trials. However, the difference in MTs between words and pseudowords remained stable even under these conditions of faster execution time, indicating a consistent slowdown in MTs for responses involving pseudowords.

The current findings solidify the conundrum concerning the nature of the decisional components shaping motor-execution processes. The dissociation in the premotor vs motor lexicality effect seems at odds with the notion of a single decision variable that continuously accumulates for the whole RT duration (e.g., Servant et al., 2021). Additionally, the dissociation of the lexicality effect on MT with respect to indexes of response control suggests that online response monitoring is not the driving force behind the lexicality effect on response execution (see also Scaltritti et al., 2024).

Albeit lacking in parsimony, one hypothesis is that additional processes may account for the slowdown in the execution of nonword responses. Nonwords are items with no stored representation in long-term memory. Decisions concerning their status may thus be influenced by a reduced confidence stemming from the lack of definitive and positive evidence for the response (Dufau et al., 2012; Grainger & Jacobs, 1996), possibly akin to more general self-terminated search processes in which no target is found. Under these premises, the lexicality effect on MTs may be linked with metacognitive confidence (e.g., Desender et al., 2021; Fleming et al., 2018) concerning the ongoing decision. Possibly, the evolving confidence variable(s) may also unfold during response execution, thus shaping MTs, in line with perspectives highlighting the involvement of the whole perception–action cycle in decision confidence (Fleming & Daw, 2017; Gajdos et al., 2019; Sanchez et al., 2024). Relatedly, without the activation of a stored representation to drive the response, the decision system may be prompted to issue a response before a definitive commitment is reached, possibly under the buildup of time-evolving urgency signals (Cisek et al., 2009), thus partially postponing the resolution of decision uncertainty during response execution. Given the results of the present experiment, we would further need to assume that lower decision confidence and/or higher uncertainty is maintained even when decision and response biases favor nonword responses. This may be due to a residual lack of positive evidence supporting nonword identification.

Indeed, the inherent uncertainty associated with nonword items has led to the hypothesis that additional processes are involved in nonword decisions with the aim to provide positive evidence concerning the non-lexical status of the item. One example is represented by late verification stages (Paap et al., 1982; Perea et al., 2005; Yap et al., 2015), during which the item is compared against the set of activated lexical candidates to detect deviations from existing words. Assuming that these late processes percolate into motor-response execution, they may exert a variable influence on MTs, depending on the degree to which the comparison provides diagnostic information about the lexical status of the item (e.g., the comparison would be particularly informative in case of pseudowords closely resembling a specific word, e.g., *elethant*).

Additionally, the elapsing time may have a specific informative value for nonword responses, as the likelihood of the stimulus being a nonword may increase over time after stimulus onset. Although classic deadline models (e.g., Coltheart et al., 2001; Grainger & Jacobs, 1996) building on this notion have shown a number of critical shortcomings (Ratcliff et al., 2004; Wagenmakers et al., 2008), more recent implementations have managed to accommodate the influence of elapsing time in nonword decisions within a leaky accumulator model (Dufau et al., 2012). Here, a nonword-response node receives excitatory input as a function of time from stimulus onset (together with an inhibitory input from the word-response node). Potentially, elapsing time may progressively drive the system towards premature activations of nonword responses with a residual part of the decision handled during response execution. Nonetheless, under these premises, the slowdown of MTs under conditions of nonword-bias would still remain unclear: If anything, in this condition, nonwords are faster than words at the level of PMTs.

To conclude, our results do not allow to provide a definitive functional definition of the MT. Nonetheless, the clear-cut dissociation of decision phenomena across the premotor and the motor components of RTs strongly points, even in the context of simple two-alternative choice tasks featuring discrete button-press response, towards multiple components in decision-making, featuring differential dynamics before and after the onset of motor activity.

## Supplementary Information

Below is the link to the electronic supplementary material.

Supplementary file1 (DOCX 32 KB)

Supplementary file2 (DOCX 14 KB)

## Data Availability

Raw data and materials are publicly available at https://osf.io/v3cx4/

## Code availability (software application or custom code)

Scripts for pre-processing and analyses are available from the last author upon request.  The data and materials are available at [https://osf.io/v3cx4/]. The experiment was not preregistered. 

## Declarations

### Open practices statement

The data and materials are available at [https://osf.io/v3cx4/]. The experiment was not preregistered.
