# Supplementary material for: Dissociating premotor and motor components of response times: Evidence of independent decisional effects during motor-response execution
Source: Psychon Bull Rev. 2025 Mar 7;32(4):1890–900. doi: 10.3758/s13423-025-02663-z (PMC12325556; doi:10.3758/s13423-025-02663-z)
Supplement: Supplementary file 1 — Supplementary file1 (DOCX 32 KB) [file 13423_2025_2663_MOESM1_ESM.docx]

**Supplementary Materials 1**

***Random Effects for (G)LMEs***

For each model of the article, we report here the random effect parameters. The pseudo-code within the table specifies the structure of fixed- and random-effects. The code *1 + effect | participant* indicates a random effect modeled as a random slope and its correlation with the random intercept, whereas *effect || participant* identifies a random slope without correlation.

**Table S1. Random effects for LMEs of chronometric measures**

| Group | Random Effect |  | Variance | SD | Correlation |  |  |  |  |
| --- | --- | --- | --- | --- | --- | --- | --- | --- | --- |
|  |  |  | RT ~ Bias * Lexicality + (1+Bias * Lexicality \| Participant) + (1 \| Item) | | | | | | |
| Items | Intercept |  | 2603.9 | 51.03 |  |  |  |  |  |
| Participants | Intercept |  | 8545.9 | 92.44 |  |  |  |  |  |
|  | Bias: PW |  | 2362.5 | 48.61 | -0.67 |  |  |  |  |
|  | Bias: W |  | 2925.2 | 54.08 | -0.72 | 0.80 |  |  |  |
|  | Lexicality (PW) |  | 2091.1 | 45.73 | 0.32 | 0.14 | 0.12 |  |  |
|  | Bias (PW) X Lex (PW) |  | 673.6 | 25.95 | -0.15 | -0.12 | 0.01 | -0.67 |  |
|  | Bias (PW) X Lex (PW) |  | 1377.8 | 37.12 | 0.33 | -0.41 | - 0.53 | -0.40 | 0.33 |
| Residual |  |  | 17364.5 | 131.77 |  |  |  |  |  |
|  |  |  | PMT ~ Bias * Lexicality + (Bias * Lexicality \|\| Participant) + (1 \| Item) | | | | | | |
| Items | Intercept |  | 2535 | 50.35 |  |  |  |  |  |
| Participants | Intercept |  | 7232.4 | 85.04 |  |  |  |  |  |
|  | Bias: PW |  | 1773.8 | 42.12 | - |  |  |  |  |
|  | Bias: W |  | 1775.1 | 42.13 | - | - |  |  |  |

**Table S1. Continues**

| Group | Random Effect |  | Variance | SD | Correlation |  |  |  |  |
| --- | --- | --- | --- | --- | --- | --- | --- | --- | --- |
|  | Lexicality (PW) |  | 1402.7 | 37.45 | - | - | - |  |  |
|  | Bias (PW) X Lex (PW) |  | 424.7 | 20.61 | - | - | - | - |  |
|  | Bias (PW) X Lex (PW) |  | 593.8 | 24.37 | - | - | - | - | - |
| Residual |  |  | 16950.2 | 130.19 |  |  |  |  |  |
|  |  |  | MT ~ Bias * Lexicality + (Bias * Lexicality \|\| Participant) + (1 \| Item) | | | | | | |
| Items | Intercept |  | 1.73 | 1.31 |  |  |  |  |  |
| Participants | Intercept |  | 582.40 | 24.13 |  |  |  |  |  |
|  | Bias: PW |  | 180.79 | 13.45 | - |  |  |  |  |
|  | Bias: W |  | 182.53 | 13.51 | - | - |  |  |  |
|  | Lexicality (PW) |  | 60.78 | 7.80 | - | - | - |  |  |
|  | Bias (PW) X Lex (PW) |  | 79.03 | 8.89 | - | - | - | - |  |
|  | Bias (PW) X Lex (PW) |  | 104.02 | 10.20 | - | - | - | - | - |
| Residual |  |  | 883.51 | 29.72 |  |  |  |  |  |

*Note.* RT = reaction time; PMT = premotor time; MT = motor time; SD = standard deviation; PW = pseudowords; W = words.

**Table S2. Random effects for GLMEs of accuracy, partial errors, and CAFs.**

| Group | Random Effect |  | Variance | SD | Correlation | |
| --- | --- | --- | --- | --- | --- | --- |
| Accuracy  ACC ~ Bias * Lexicality + (Bias * Lexicality \| \| Participant) + (1 \| Item) | | | | | | |
| Item | Intercept |  | 1.25 | 1.12 |  | |
| Participant | Intercept |  | 0.57 | 0.76 |  | |
|  | Bias: PW |  | 0.09 | 0.30 |  | |
|  | Bias: W |  | 0.03 | 0.17 |  | |
|  | Lexicality (PW) |  | 0.18 | 0.43 |  | |
|  | Bias (PW) X Lex (PW) |  | 0.04 | 0.22 |  | |
|  | Bias (PW) X Lex (PW) |  | 0.11 | 0.34 |  | |
| Partial Error ~ Bias * Lexicality + (Bias * Lexicality \| \| Participant) + (1 \| Item) | | | | | | |
| Item | Intercept |  | 0.27 | 0.52 |  | |
| Participant | Intercept |  | 0.72 | 0.85 |  | |
|  | Bias: PW |  | 0.09 | 0.31 |  | |
|  | Bias: W |  | 0.08 | 0.29 |  |  |
|  | Lexicality (PW) |  | 0.07 | 0.26 |  |  |
|  | Bias (PW) X Lex (PW) |  | 0.06 | 0.24 |  |  |
|  | Bias (PW) X Lex (PW) |  | 0.07 | 0.26 |  |  |
| CAF  ACC ~ Bias * Lexicality * poly(Quant, 2) + (1 \| Participant) + (1 \| Item) | | | | | | |
| Item | Intercept |  | 1.73 | 1.32 |  |  |
| Participant | Intercept |  | 0.68 | 0.83 |  |  |

*Note.* ACC = accuracy; PE = partial error; SD = standard deviation.
