# Supplementary material for: Dissociating premotor and motor components of response times: Evidence of independent decisional effects during motor-response execution
Source: Psychon Bull Rev. 2025 Mar 7;32(4):1890–900. doi: 10.3758/s13423-025-02663-z (PMC12325556; doi:10.3758/s13423-025-02663-z)
Supplement: Supplementary file 2 — Supplementary file2 (DOCX 14 KB) [file 13423_2025_2663_MOESM2_ESM.docx]

**Appendix A**

**Inferential problems Set 1 (unlimited time)**

**Consistent inferences**

All dogs have legs. Fish do not have legs. Conclusion: Fish are not dogs.

All trees have roots. Radios do not have roots. Conclusion: Radios are not trees.

All desserts are sweet. Hats are not sweet. Conclusion: Hats are not desserts.

All things that fly in the sky have lungs. Cadavers have lungs. Conclusion: Cadavers fly in the sky.

All trees have leaves. Tulips have leaves. Conclusion: Tulips are trees.

All animals have hairs. Carpets have hairs. Conclusion: Carpets are animals.

**Conflict inferences**

All dogs have legs. German Shepherds have legs. Conclusion: German Shepherds are dogs.

All trees have roots. Maple trees have roots. Conclusion: Maple trees are trees.

All desserts are sweet. Cakes are sweet. Conclusion: Cakes are desserts.

All things that fly in the sky have lungs. Airplanes do not have lungs. Conclusion: Airplanes do not fly in the sky.

All trees have leaves. Fir trees do not have leaves. Conclusion: Fir trees are not trees.

All animals have hairs. Birds do not have hairs. Conclusion: Birds are not animals.

**Inferential problems Set 2 (4s time)**

**Consistent inferences**

All birds fly. Cows do not fly. Conclusion: Cows are not birds.

All fruits are sweet. Broccoli is not sweet. Conclusion: Broccoli is not a fruit.

All trees have leaves. Radios do not have leaves. Conclusion: Radios are not trees.

All humans breathe. Birds breathe. Conclusion: Birds are human.

All automobiles have motors. Airplanes have motors. Conclusion: Airplanes are automobiles.

All food has a price. Gasoline has a price. Conclusion: Gasoline is a food.

**Conflict inferences**

All birds fly. Ostriches do not fly. Conclusion: Ostriches are not birds.

All fruits are sweet. Lemons are not sweet. Conclusion: Lemons are not fruit.

All trees have leaves. Fir trees do not have leaves. Conclusion: Fir trees are not trees.

All humans breathe. Italians breathe. Conclusion: Italians are human.

All vehicles have motors. Automobiles have motors. Conclusion: Automobiles are vehicles.

All foods have a price. Apples have a price. Conclusion: Apples are food.
